# Author’s Reply: Extending section 12 approval under the Mental Health Act to professions other than medicine

**DOI:** 10.1192/bjb.2025.30

**Published:** 2025-06

**Authors:** John L. Taylor

**Affiliations:** Northumbria Law School, Northumbria University, Newcastle, UK

## The issue is a lack of internal logic in the Mental Health Act 1983 section 12 eligibility criteria

As the husband of a retired general practitioner (GP), I understand and concur with Claire Hilton’s and Alan Cohen’s points about the impact of successive National Health Service policy developments on the clinical practice of GPs and their ability to build long-term relationships with patients on their lists. However, this was not the point of my editorial with Carole Burrell.

Our point concerned the lack of internal logic in the Mental Health Act 1983, which, under section 12, automatically approves medical practitioners who have been approved as approved clinicians by regional approvals panels on the basis of their having the required ‘special experience in the diagnosis or treatment of mental disorder’, while excluding approved clinicians who are not medical practitioners from section 12 approval. We contrasted this anomaly with the status quo concerning GPs who, despite the issues outlined by Hilton and Cohen, continue to be eligible for section 12 approval without the ‘special experience’ required in the primary legislation. Our editorial concerned this inconsistency in the law and its impact on practice in a modern mental health service, not the working conditions of and disaffection among GPs, which are of course important nonetheless.

Although the Wessely review did not refer to section 12 eligibility criteria specifically, it did highlight significant issues with timely access to section 12 doctors and the impact on patients of undue delays in Mental Health Act assessments being completed, and it recommended that this issue should be addressed and reviewed. Our proposals will assist with remedying this situation for the benefit of patients – as well as correcting a clear anomaly in the law.

The Mental Health Bill 2025 is now proceeding through parliament, and the issue of section 12 approval has been subject to a proposed amendment at the House of Lords committee stage. It is anticipated that this issue will be debated further when the Bill reaches the House of Commons in the near future. It would be beneficial for all involved – including GPs, but especially patients – if section 12 approval criteria were amended to include all approved clinicians.

